# *N*-(2,4-Dimethyl­phen­yl)-2,2-diphenyl­acetamide

**DOI:** 10.1107/S1600536812014079

**Published:** 2012-04-06

**Authors:** Hoong-Kun Fun, Tze Shyang Chia, Prakash S. Nayak, B. Narayana, B. K. Sarojini

**Affiliations:** a X-ray Crystallography Unit, School of Physics, Universiti Sains Malaysia, 11800 USM, Penang, Malaysia; b Department of Studies in Chemistry, Mangalore University, Mangalagangotri 574 199, India; c Department of Chemistry, P.A. College of Engineering, Nadupadavu, Montepadavu, PO, Mangalore 574 153, India

## Abstract

The asymmetric unit of the title compound, C_22_H_21_NO, consists of two crystallographically independent mol­ecules (*A* and *B*). Each mol­ecule contains two benzene rings and one dimethyl­benzene ring. The dihedral angle between the two benzene rings is 87.75 (16)° in mol­ecule *A* and 89.25 (16)° in mol­ecule *B*. In mol­ecule *A*, the dimethyl­benzene ring forms dihedral angles of 89.65 (8) and 42.98 (11)° with the two benzene rings, whereas the corresponding angles are equal to 63.15 (7) and 58.67 (10)° in mol­ecule *B*. An intra­molecular C—H⋯O hydrogen bond generates an *S*(6) ring motif in each mol­ecule. In the crystal, mol­ecules are linked by bifurcated (N,C)—H⋯O hydrogen bonds, generating *R*_2_^1^(6) ring motifs and forming infinite chains along the *a* axis. The crystal is further stabilized by C—H⋯π and π–π inter­actions with centroid–centroid distances of 3.8543 (18) and 3.930 (2) Å.

## Related literature

For the structural similarity of *N*-substituted 2-aryl­acetamides to the lateral chain of natural benzyl­penicillin, see: Mijin & Marinkovic (2006 ▶); Mijin *et al.* (2008 ▶). For the coordination abilities of amides, see: Wu *et al.* (2008 ▶, 2010 ▶). For related structures, see: Praveen *et al.* (2011*a* ▶,*b* ▶,*c* ▶); Fun *et al.* (2011*a* ▶,*b* ▶). For hydrogen-bond motifs, see: Bernstein *et al.* (1995 ▶). For reference bond lengths, see: Allen *et al.* (1987 ▶).
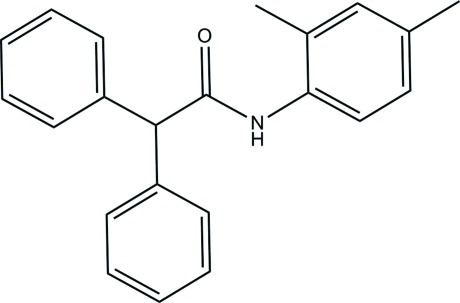


## Experimental

### 

#### Crystal data

C_22_H_21_NO
*M* *_r_* = 315.40
Triclinic, 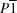

*a* = 9.5520 (9) Å
*b* = 10.2011 (10) Å
*c* = 17.9656 (17) Å
α = 91.030 (2)°
β = 98.957 (2)°
γ = 90.377 (2)°
*V* = 1728.9 (3) Å^3^
*Z* = 4
Mo *K*α radiation
μ = 0.07 mm^−1^
*T* = 100 K
0.41 × 0.19 × 0.17 mm

#### Data collection

Bruker APEX DUO CCD area-detector diffractometer
Absorption correction: multi-scan (*SADABS*; Bruker, 2009 ▶) *T*_min_ = 0.970, *T*_max_ = 0.987
30869 measured reflections
7858 independent reflections
6570 reflections with *I* > 2σ(*I*)
*R*_int_ = 0.040

#### Refinement

*R*[*F* ^2^ > 2σ(*F* ^2^)] = 0.085
*wR*(*F* ^2^) = 0.261
*S* = 1.14
7858 reflections
445 parameters
H atoms treated by a mixture of independent and constrained refinement
Δρ_max_ = 0.61 e Å^−3^
Δρ_min_ = −0.42 e Å^−3^

### 

Data collection: *APEX2* (Bruker, 2009 ▶); cell refinement: *SAINT* (Bruker, 2009 ▶); data reduction: *SAINT*; program(s) used to solve structure: *SHELXTL* (Sheldrick, 2008 ▶); program(s) used to refine structure: *SHELXTL*; molecular graphics: *SHELXTL*; software used to prepare material for publication: *SHELXTL* and *PLATON* (Spek, 2009 ▶).

## Supplementary Material

Crystal structure: contains datablock(s) global, I. DOI: 10.1107/S1600536812014079/ez2289sup1.cif

Structure factors: contains datablock(s) I. DOI: 10.1107/S1600536812014079/ez2289Isup2.hkl

Supplementary material file. DOI: 10.1107/S1600536812014079/ez2289Isup3.cml

Additional supplementary materials:  crystallographic information; 3D view; checkCIF report

## Figures and Tables

**Table 1:** Hydrogen-bond geometry (Å, °) *Cg*1, *Cg*4 and *Cg*5 are the centroids of the C1*A*–C6*A*, C1*B*–C6*B* and C8*B*–C13*B* rings, respectively.

*D*—H⋯*A*	*D*—H	H⋯*A*	*D*⋯*A*	*D*—H⋯*A*
N1*A*—H1*NA*⋯O1*B*^i^	0.88 (4)	2.13 (4)	2.980 (3)	163 (3)
N1*B*—H1*NB*⋯O1*A*	0.86 (5)	2.18 (5)	3.012 (3)	162 (3)
C7*A*—H7*AA*⋯O1*B*^i^	1.00	2.32	3.258 (4)	155
C9*A*—H9*AA*⋯O1*A*	0.95	2.48	3.110 (4)	123
C7*B*—H7*BA*⋯O1*A*	1.00	2.27	3.225 (4)	160
C13*B*—H13*B*⋯O1*B*	0.95	2.46	3.087 (4)	124
C3*A*—H3*AA*⋯*Cg*5^ii^	0.95	2.89	3.754 (4)	151
C4*A*—H4*AA*⋯*Cg*4	0.95	2.84	3.539 (4)	131
C13*A*—H13*A*⋯*Cg*4^i^	0.95	2.68	3.561 (3)	154
C9*B*—H9*BA*⋯*Cg*1	0.95	2.62	3.546 (3)	167

Symmetry codes: (i) 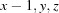; (ii) 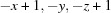.

**Fig. 1.**
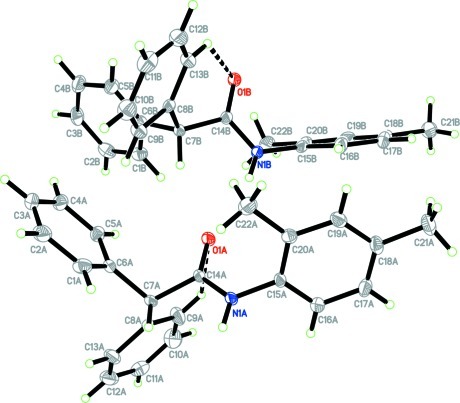
The molecular structure of the title compound with atom labels with 50% probability displacement ellipsoids. Intramolecular hydrogen bonds are shown by dashed lines.

**Fig. 2.**
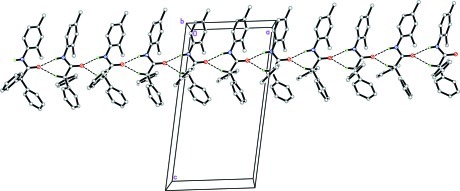
The crystal packing of the title compound. The dashed lines represent the hydrogen bonds.

## supplementary crystallographic information

### Comment

*N*-Substituted 2-arylacetamides are very interesting compounds because of their structural similarity to the lateral chain of natural benzylpenicillin (Mijin & Marinkovic, 2006; Mijin *et al.*, 2008). Amides are also used as ligands due to their excellent coordination abilities (Wu *et al.*, 2008, 2010). Crystal structures of some acetamide derivatives *viz.*, *N*-(4-chloro-1,3-benzothiazol-2-yl)-2-(3-methylphenyl)acetamide monohydrate, *N*-(3-chloro-4-fluorophenyl)-2,2-diphenylacetamide and *N*-(3-chloro-4-fluorophenyl)-2-(naphthalen-1-yl)acetamide (Praveen *et al.*, 2011*a*,*b*,*c*) have been reported. In continuation of our work on synthesis of amides (Fun *et al.*, 2011*a*,*b*), we report herein the crystal structure of the title compound (I).

The asymmetric unit of the title compound consists of two crystallographically independent molecules (*A* and *B*) as shown in Fig. 1. Each molecule contains two benzene rings (C1–C6 & C8–C13) and one dimethylbenzene ring (C15–C22) [maximum deviation = 0.0185 (27) Å at atom C19A in molecule *A* and 0.0155 (21) Å at atom C21B in molecule *B*]. The dihedral angle between the two benzene rings is 87.75 (16)° in molecule *A* and 89.25 (16)° in molecule *B*. In molecule *A*, the dimethylbenzene ring forms dihedral angles of 89.65 (8) and 42.98 (11)° with the two benzene rings (C1–C6 and C8–C13 respectively), whereas the corresponding angles are equal to 63.15 (7) and 58.67 (10)° in molecule *B*. Bond lengths (Allen *et al.*, 1987) and angles are within normal ranges and are comparable to related structures (Fun *et al.*, 2011*a*,*b*). Intramolecular C9A—H9AA···O1A hydrogen bond (Table 1) generates an S(6) ring motif (Fig. 1; Bernstein *et al.*, 1995) in molecule *A*, whereas the same ring motif is generated by C13B—H13B···O1B hydrogen bonds in molecule *B*.

In the crystal, molecules are linked by intermolecular bifurcated N1A—H1NA···O1B, N1B—H1NB···O1A, C7A—H7AA···O1B and C7B—H7BA···O1A hydrogen bonds (Table 1), generating *R*_2_^1^(6) ring motifs and forming infinite chains along the *a* axis. The crystal is further stabilized by C—H···π interactions, involving *Cg*1, *Cg*4 and *Cg*5 which are the centroids of C1A–C6A, C1B–C6B and C8B–C13B rings, respectively. π—π interactions are also observed with *Cg*3···*Cg*3 and *Cg*6···*Cg*6 distances of 3.8543 (18) Å[symmetry code: –*X*,-Y,-*Z*] and 3.930 (2) Å[symmetry code: 1-*X*,1-Y,-*Z*], where *Cg*3 and *Cg*6 are the centroids of C15A–C20A and C15B–C20B, respectively.

### Experimental

Diphenylacetic acid (0.212 g, 1 mmol), 2,4-dimethylaniline (0.1 ml, 1 mmol) and 1-ethyl-3-(3-dimethylaminopropyl)-carbodiimide hydrochloride (1.0 g, 0.01 mol) were dissolved in dichloromethane (20 ml). The mixture was stirred in the presence of triethylamine at 273 K for about 3 h. The contents were poured into 100 ml of ice-cold aqueous hydrochloric acid with stirring which was then extracted thrice with dichloromethane. Organic layer was washed with saturated NaHCO_3_ solution and brine solution, dried and concentrated under reduced pressure to give the title compound (I). Single crystals were grown from methylene chloride and ethanol (1:1) mixture by the slow evaporation method (M.P.: 430–432 K).

### Refinement

Atom H1NA and H1NB were located from difference fourier map and refined freely [N—H = 0.88 (4) and 0.86 (5) Å]. The remaining H atoms were positioned geometrically [C—H = 0.95, 0.98 and 1.00 Å] and refined using a riding model with *U*_iso_(H) = 1.2 or 1.5*U*_eq_(C). A rotating group model was applied to the methyl group. Three outliers (-3 1 3), (4 - 1 6) and (1 - 1 13) were omitted.

### Crystal data

**Table:**

C_22_H_21_NO	*Z* = 4
*M**_r_* = 315.40	*F*(000) = 672
Triclinic, *P*1	*D*_x_ = 1.212 Mg m^−^^3^
Hall symbol: -P 1	Mo *K*α radiation, λ = 0.71073 Å
*a* = 9.5520 (9) Å	Cell parameters from 9946 reflections
*b* = 10.2011 (10) Å	θ = 2.3–32.3°
*c* = 17.9656 (17) Å	µ = 0.07 mm^−^^1^
α = 91.030 (2)°	*T* = 100 K
β = 98.957 (2)°	Block, colourless
γ = 90.377 (2)°	0.41 × 0.19 × 0.17 mm
*V* = 1728.9 (3) Å^3^	

### Data collection

**Table:**

Bruker APEX DUO CCD area-detector diffractometer	7858 independent reflections
Radiation source: fine-focus sealed tube	6570 reflections with *I* > 2σ(*I*)
Graphite monochromator	*R*_int_ = 0.040
φ and ω scans	θ_max_ = 27.5°, θ_min_ = 2.0°
Absorption correction: multi-scan (*SADABS*; Bruker, 2009)	*h* = −12→12
*T*_min_ = 0.970, *T*_max_ = 0.987	*k* = −13→13
30869 measured reflections	*l* = −23→23

### Refinement

**Table:**

Refinement on *F*^2^	Primary atom site location: structure-invariant direct methods
Least-squares matrix: full	Secondary atom site location: difference Fourier map
*R*[*F*^2^ > 2σ(*F*^2^)] = 0.085	Hydrogen site location: inferred from neighbouring sites
*wR*(*F*^2^) = 0.261	H atoms treated by a mixture of independent and constrained refinement
*S* = 1.14	*w* = 1/[σ^2^(*F*_o_^2^) + (0.097*P*)^2^ + 5.029*P*] where *P* = (*F*_o_^2^ + 2*F*_c_^2^)/3
7858 reflections	(Δ/σ)_max_ < 0.001
445 parameters	Δρ_max_ = 0.61 e Å^−^^3^
0 restraints	Δρ_min_ = −0.42 e Å^−^^3^

### Special details

**Table:**

Experimental. The crystal was placed in the cold stream of an Oxford Cryosystems Cobra open-flow nitrogen cryostat (Cosier & Glazer, 1986) operating at 100.0 (1) K.
Geometry. All e.s.d.'s (except the e.s.d. in the dihedral angle between two l.s. planes) are estimated using the full covariance matrix. The cell e.s.d.'s are taken into account individually in the estimation of e.s.d.'s in distances, angles and torsion angles; correlations between e.s.d.'s in cell parameters are only used when they are defined by crystal symmetry. An approximate (isotropic) treatment of cell e.s.d.'s is used for estimating e.s.d.'s involving l.s. planes.
Refinement. Refinement of *F*^2^ against ALL reflections. The weighted *R*-factor *wR* and goodness of fit *S* are based on *F*^2^, conventional *R*-factors *R* are based on *F*, with *F* set to zero for negative *F*^2^. The threshold expression of *F*^2^ > σ(*F*^2^) is used only for calculating *R*-factors(gt) *etc*. and is not relevant to the choice of reflections for refinement. *R*-factors based on *F*^2^ are statistically about twice as large as those based on *F*, and *R*- factors based on ALL data will be even larger.

### Fractional atomic coordinates and isotropic or equivalent isotropic displacement parameters (Å^2^)

**Table:**

	*x*	*y*	*z*	*U*_iso_*/*U*_eq_	
O1A	0.2422 (2)	0.2899 (2)	0.22082 (12)	0.0202 (5)	
N1A	0.0206 (3)	0.2282 (3)	0.16612 (14)	0.0173 (5)	
C1A	0.0812 (3)	0.1366 (3)	0.3930 (2)	0.0233 (6)	
H1AA	−0.0062	0.0995	0.3692	0.028*	
C2A	0.1554 (4)	0.0785 (3)	0.4571 (2)	0.0281 (7)	
H2AA	0.1171	0.0032	0.4772	0.034*	
C3A	0.2845 (4)	0.1301 (3)	0.49134 (18)	0.0255 (7)	
H3AA	0.3344	0.0902	0.5349	0.031*	
C4A	0.3407 (4)	0.2397 (3)	0.46204 (18)	0.0226 (6)	
H4AA	0.4301	0.2743	0.4848	0.027*	
C5A	0.2655 (3)	0.2991 (3)	0.39907 (17)	0.0190 (6)	
H5AA	0.3033	0.3753	0.3796	0.023*	
C6A	0.1347 (3)	0.2479 (3)	0.36411 (16)	0.0163 (6)	
C7A	0.0518 (3)	0.3118 (3)	0.29485 (16)	0.0159 (6)	
H7AA	−0.0467	0.2749	0.2878	0.019*	
C8A	0.0405 (3)	0.4598 (3)	0.30493 (16)	0.0166 (6)	
C9A	0.1067 (4)	0.5504 (3)	0.26542 (19)	0.0271 (7)	
H9AA	0.1658	0.5209	0.2307	0.033*	
C10A	0.0877 (4)	0.6843 (4)	0.2758 (2)	0.0321 (8)	
H10A	0.1340	0.7454	0.2484	0.039*	
C11A	0.0020 (4)	0.7286 (3)	0.3260 (2)	0.0273 (7)	
H11A	−0.0126	0.8199	0.3323	0.033*	
C12A	−0.0624 (4)	0.6392 (3)	0.3667 (2)	0.0289 (7)	
H12A	−0.1192	0.6694	0.4024	0.035*	
C13A	−0.0448 (3)	0.5061 (3)	0.35622 (19)	0.0238 (7)	
H13A	−0.0911	0.4455	0.3840	0.029*	
C14A	0.1152 (3)	0.2754 (3)	0.22425 (16)	0.0153 (5)	
C15A	0.0619 (3)	0.1939 (3)	0.09481 (16)	0.0173 (6)	
C16A	0.0185 (3)	0.2723 (3)	0.03354 (17)	0.0218 (6)	
H16A	−0.0384	0.3469	0.0389	0.026*	
C17A	0.0588 (3)	0.2413 (3)	−0.03656 (18)	0.0247 (7)	
H17A	0.0294	0.2954	−0.0785	0.030*	
C18A	0.1409 (3)	0.1327 (3)	−0.04488 (17)	0.0224 (6)	
C19A	0.1815 (3)	0.0544 (3)	0.01743 (18)	0.0215 (6)	
H19A	0.2365	−0.0213	0.0115	0.026*	
C20A	0.1447 (3)	0.0825 (3)	0.08783 (17)	0.0188 (6)	
C21A	0.1879 (4)	0.0999 (4)	−0.11911 (19)	0.0303 (8)	
H21A	0.1206	0.1363	−0.1602	0.045*	
H21B	0.1912	0.0044	−0.1257	0.045*	
H21C	0.2823	0.1375	−0.1198	0.045*	
C22A	0.1939 (4)	−0.0053 (3)	0.15325 (19)	0.0258 (7)	
H22A	0.2083	−0.0941	0.1343	0.039*	
H22B	0.1220	−0.0078	0.1866	0.039*	
H22C	0.2832	0.0288	0.1813	0.039*	
O1B	0.7412 (2)	0.1999 (2)	0.21860 (12)	0.0192 (5)	
N1B	0.5215 (3)	0.2611 (3)	0.16445 (14)	0.0171 (5)	
C1B	0.5909 (3)	0.3858 (3)	0.36734 (19)	0.0216 (6)	
H1BA	0.5300	0.4273	0.3280	0.026*	
C2B	0.6464 (4)	0.4580 (3)	0.4319 (2)	0.0250 (7)	
H2BA	0.6213	0.5472	0.4370	0.030*	
C3B	0.7388 (4)	0.3989 (3)	0.48871 (18)	0.0259 (7)	
H3BA	0.7778	0.4475	0.5327	0.031*	
C4B	0.7732 (4)	0.2688 (3)	0.48044 (18)	0.0255 (7)	
H4BA	0.8368	0.2284	0.5190	0.031*	
C5B	0.7163 (3)	0.1959 (3)	0.41661 (18)	0.0215 (6)	
H5BA	0.7411	0.1065	0.4120	0.026*	
C6B	0.6232 (3)	0.2537 (3)	0.35958 (15)	0.0155 (5)	
C7B	0.5484 (3)	0.1774 (3)	0.29080 (16)	0.0149 (5)	
H7BA	0.4486	0.2093	0.2816	0.018*	
C8B	0.5398 (3)	0.0301 (3)	0.30118 (15)	0.0155 (5)	
C9B	0.4277 (3)	−0.0183 (3)	0.33470 (18)	0.0209 (6)	
H9BA	0.3620	0.0409	0.3511	0.025*	
C10B	0.4114 (4)	−0.1520 (3)	0.3442 (2)	0.0267 (7)	
H10B	0.3354	−0.1835	0.3675	0.032*	
C11B	0.5055 (4)	−0.2397 (3)	0.3198 (2)	0.0277 (7)	
H11B	0.4936	−0.3313	0.3257	0.033*	
C12B	0.6170 (4)	−0.1927 (3)	0.28682 (19)	0.0275 (7)	
H12B	0.6817	−0.2526	0.2700	0.033*	
C13B	0.6355 (3)	−0.0590 (3)	0.27790 (18)	0.0220 (6)	
H13B	0.7134	−0.0281	0.2559	0.026*	
C14B	0.6141 (3)	0.2129 (3)	0.22130 (16)	0.0150 (5)	
C15B	0.5644 (3)	0.3007 (3)	0.09483 (16)	0.0175 (6)	
C16B	0.5224 (3)	0.2252 (3)	0.03030 (17)	0.0221 (6)	
H16B	0.4661	0.1488	0.0326	0.027*	
C17B	0.5623 (4)	0.2607 (4)	−0.03800 (18)	0.0267 (7)	
H17B	0.5340	0.2078	−0.0819	0.032*	
C18B	0.6432 (3)	0.3733 (4)	−0.04225 (18)	0.0249 (7)	
C19B	0.6823 (3)	0.4486 (3)	0.02270 (19)	0.0229 (6)	
H19B	0.7368	0.5261	0.0198	0.028*	
C20B	0.6451 (3)	0.4153 (3)	0.09239 (17)	0.0198 (6)	
C21B	0.6900 (4)	0.4120 (4)	−0.1154 (2)	0.0352 (9)	
H21D	0.6675	0.5043	−0.1251	0.053*	
H21E	0.6405	0.3572	−0.1567	0.053*	
H21F	0.7925	0.3996	−0.1118	0.053*	
C22B	0.6906 (4)	0.5003 (3)	0.16090 (19)	0.0266 (7)	
H22D	0.7101	0.5894	0.1454	0.040*	
H22E	0.7766	0.4645	0.1901	0.040*	
H22F	0.6149	0.5028	0.1920	0.040*	
H1NA	−0.069 (4)	0.224 (3)	0.172 (2)	0.015 (8)*	
H1NB	0.434 (5)	0.262 (4)	0.171 (2)	0.030 (11)*	

### Atomic displacement parameters (Å^2^)

**Table:**

	*U*^11^	*U*^22^	*U*^33^	*U*^12^	*U*^13^	*U*^23^
O1A	0.0105 (9)	0.0352 (12)	0.0149 (10)	−0.0014 (8)	0.0022 (8)	−0.0017 (8)
N1A	0.0088 (11)	0.0310 (14)	0.0120 (12)	−0.0012 (9)	0.0017 (9)	−0.0047 (10)
C1A	0.0153 (14)	0.0243 (15)	0.0304 (17)	−0.0004 (11)	0.0034 (12)	0.0034 (13)
C2A	0.0234 (16)	0.0298 (17)	0.0338 (19)	0.0040 (13)	0.0117 (14)	0.0122 (14)
C3A	0.0277 (16)	0.0323 (17)	0.0171 (15)	0.0087 (13)	0.0046 (12)	0.0042 (12)
C4A	0.0251 (15)	0.0241 (15)	0.0176 (15)	0.0040 (12)	0.0006 (12)	−0.0024 (11)
C5A	0.0214 (14)	0.0184 (14)	0.0167 (14)	0.0007 (11)	0.0014 (11)	−0.0012 (11)
C6A	0.0148 (13)	0.0218 (14)	0.0131 (13)	0.0024 (10)	0.0044 (10)	−0.0005 (10)
C7A	0.0084 (12)	0.0256 (15)	0.0141 (13)	−0.0003 (10)	0.0030 (10)	−0.0006 (11)
C8A	0.0093 (12)	0.0274 (15)	0.0121 (13)	0.0011 (10)	−0.0013 (10)	0.0014 (11)
C9A	0.0295 (17)	0.0312 (17)	0.0234 (16)	−0.0005 (13)	0.0125 (13)	0.0030 (13)
C10A	0.037 (2)	0.0266 (17)	0.0335 (19)	−0.0049 (14)	0.0076 (15)	0.0053 (14)
C11A	0.0243 (16)	0.0240 (16)	0.0308 (18)	0.0018 (12)	−0.0049 (13)	−0.0005 (13)
C12A	0.0221 (16)	0.0320 (18)	0.0328 (19)	0.0059 (13)	0.0052 (14)	−0.0023 (14)
C13A	0.0194 (15)	0.0280 (16)	0.0262 (16)	0.0025 (12)	0.0099 (12)	0.0001 (13)
C14A	0.0114 (12)	0.0238 (14)	0.0109 (13)	0.0005 (10)	0.0024 (10)	−0.0002 (10)
C15A	0.0117 (12)	0.0286 (15)	0.0114 (13)	−0.0021 (11)	0.0014 (10)	−0.0041 (11)
C16A	0.0192 (14)	0.0293 (16)	0.0163 (14)	0.0028 (12)	0.0016 (11)	−0.0028 (12)
C17A	0.0241 (16)	0.0349 (18)	0.0139 (14)	−0.0017 (13)	−0.0004 (12)	−0.0003 (12)
C18A	0.0176 (14)	0.0345 (17)	0.0153 (14)	−0.0078 (12)	0.0043 (11)	−0.0069 (12)
C19A	0.0156 (14)	0.0268 (16)	0.0221 (15)	−0.0006 (11)	0.0040 (11)	−0.0063 (12)
C20A	0.0120 (13)	0.0260 (15)	0.0178 (14)	−0.0023 (11)	0.0013 (10)	−0.0022 (11)
C21A	0.0262 (17)	0.046 (2)	0.0197 (16)	−0.0060 (15)	0.0089 (13)	−0.0096 (14)
C22A	0.0242 (16)	0.0276 (16)	0.0244 (16)	0.0021 (12)	0.0004 (13)	0.0010 (13)
O1B	0.0102 (9)	0.0326 (12)	0.0149 (10)	−0.0001 (8)	0.0023 (7)	0.0016 (8)
N1B	0.0094 (11)	0.0297 (14)	0.0125 (12)	0.0004 (9)	0.0027 (9)	0.0034 (9)
C1B	0.0173 (14)	0.0231 (15)	0.0239 (16)	0.0006 (11)	0.0021 (11)	0.0009 (12)
C2B	0.0233 (16)	0.0210 (15)	0.0322 (18)	−0.0007 (12)	0.0101 (13)	−0.0050 (13)
C3B	0.0305 (17)	0.0308 (17)	0.0159 (15)	−0.0051 (13)	0.0035 (12)	−0.0064 (12)
C4B	0.0273 (16)	0.0314 (17)	0.0167 (15)	−0.0003 (13)	0.0001 (12)	0.0023 (12)
C5B	0.0246 (15)	0.0203 (14)	0.0192 (15)	0.0018 (12)	0.0018 (12)	0.0007 (11)
C6B	0.0135 (12)	0.0234 (14)	0.0105 (12)	−0.0017 (10)	0.0052 (10)	−0.0014 (10)
C7B	0.0110 (12)	0.0216 (14)	0.0123 (13)	0.0011 (10)	0.0023 (10)	−0.0002 (10)
C8B	0.0115 (12)	0.0228 (14)	0.0111 (12)	−0.0001 (10)	−0.0013 (10)	−0.0003 (10)
C9B	0.0186 (14)	0.0240 (15)	0.0211 (15)	0.0008 (11)	0.0061 (11)	0.0002 (11)
C10B	0.0252 (16)	0.0282 (17)	0.0272 (17)	−0.0048 (13)	0.0052 (13)	0.0055 (13)
C11B	0.0344 (18)	0.0190 (15)	0.0271 (17)	−0.0022 (13)	−0.0029 (14)	0.0004 (12)
C12B	0.0305 (17)	0.0274 (17)	0.0241 (16)	0.0080 (13)	0.0029 (13)	−0.0039 (13)
C13B	0.0201 (15)	0.0276 (16)	0.0191 (15)	0.0040 (12)	0.0058 (11)	0.0001 (12)
C14B	0.0122 (12)	0.0200 (14)	0.0125 (13)	−0.0011 (10)	0.0010 (10)	−0.0003 (10)
C15B	0.0101 (12)	0.0298 (16)	0.0128 (13)	0.0018 (11)	0.0016 (10)	0.0045 (11)
C16B	0.0160 (14)	0.0330 (17)	0.0165 (14)	−0.0026 (12)	−0.0001 (11)	0.0015 (12)
C17B	0.0236 (16)	0.0416 (19)	0.0141 (14)	0.0036 (13)	0.0005 (12)	0.0005 (13)
C18B	0.0183 (14)	0.0414 (19)	0.0167 (14)	0.0093 (13)	0.0068 (11)	0.0108 (13)
C19B	0.0136 (13)	0.0318 (17)	0.0242 (16)	0.0019 (11)	0.0042 (11)	0.0096 (13)
C20B	0.0140 (13)	0.0276 (16)	0.0176 (14)	0.0014 (11)	0.0015 (11)	0.0043 (11)
C21B	0.0275 (18)	0.059 (2)	0.0227 (17)	0.0099 (16)	0.0135 (14)	0.0159 (16)
C22B	0.0272 (17)	0.0266 (16)	0.0249 (17)	−0.0036 (13)	0.0009 (13)	0.0005 (13)

### Geometric parameters (Å, º)

**Table:**

O1A—C14A	1.233 (3)	O1B—C14B	1.231 (3)
N1A—C14A	1.350 (4)	N1B—C14B	1.345 (4)
N1A—C15A	1.436 (4)	N1B—C15B	1.440 (4)
N1A—H1NA	0.88 (4)	N1B—H1NB	0.86 (5)
C1A—C6A	1.384 (4)	C1B—C2B	1.393 (5)
C1A—C2A	1.398 (5)	C1B—C6B	1.394 (4)
C1A—H1AA	0.9500	C1B—H1BA	0.9500
C2A—C3A	1.385 (5)	C2B—C3B	1.390 (5)
C2A—H2AA	0.9500	C2B—H2BA	0.9500
C3A—C4A	1.385 (5)	C3B—C4B	1.380 (5)
C3A—H3AA	0.9500	C3B—H3BA	0.9500
C4A—C5A	1.393 (4)	C4B—C5B	1.390 (4)
C4A—H4AA	0.9500	C4B—H4BA	0.9500
C5A—C6A	1.401 (4)	C5B—C6B	1.391 (4)
C5A—H5AA	0.9500	C5B—H5BA	0.9500
C6A—C7A	1.526 (4)	C6B—C7B	1.525 (4)
C7A—C8A	1.523 (4)	C7B—C8B	1.521 (4)
C7A—C14A	1.529 (4)	C7B—C14B	1.530 (4)
C7A—H7AA	1.0000	C7B—H7BA	1.0000
C8A—C9A	1.383 (4)	C8B—C13B	1.397 (4)
C8A—C13A	1.400 (4)	C8B—C9B	1.399 (4)
C9A—C10A	1.392 (5)	C9B—C10B	1.388 (5)
C9A—H9AA	0.9500	C9B—H9BA	0.9500
C10A—C11A	1.381 (5)	C10B—C11B	1.385 (5)
C10A—H10A	0.9500	C10B—H10B	0.9500
C11A—C12A	1.380 (5)	C11B—C12B	1.385 (5)
C11A—H11A	0.9500	C11B—H11B	0.9500
C12A—C13A	1.383 (5)	C12B—C13B	1.389 (5)
C12A—H12A	0.9500	C12B—H12B	0.9500
C13A—H13A	0.9500	C13B—H13B	0.9500
C15A—C16A	1.385 (4)	C15B—C16B	1.385 (4)
C15A—C20A	1.404 (4)	C15B—C20B	1.401 (4)
C16A—C17A	1.404 (4)	C16B—C17B	1.393 (4)
C16A—H16A	0.9500	C16B—H16B	0.9500
C17A—C18A	1.381 (5)	C17B—C18B	1.390 (5)
C17A—H17A	0.9500	C17B—H17B	0.9500
C18A—C19A	1.394 (5)	C18B—C19B	1.385 (5)
C18A—C21A	1.505 (4)	C18B—C21B	1.511 (4)
C19A—C20A	1.390 (4)	C19B—C20B	1.401 (4)
C19A—H19A	0.9500	C19B—H19B	0.9500
C20A—C22A	1.508 (4)	C20B—C22B	1.499 (4)
C21A—H21A	0.9800	C21B—H21D	0.9800
C21A—H21B	0.9800	C21B—H21E	0.9800
C21A—H21C	0.9800	C21B—H21F	0.9800
C22A—H22A	0.9800	C22B—H22D	0.9800
C22A—H22B	0.9800	C22B—H22E	0.9800
C22A—H22C	0.9800	C22B—H22F	0.9800
			
C14A—N1A—C15A	121.6 (2)	C14B—N1B—C15B	122.0 (2)
C14A—N1A—H1NA	118 (2)	C14B—N1B—H1NB	116 (3)
C15A—N1A—H1NA	120 (2)	C15B—N1B—H1NB	122 (3)
C6A—C1A—C2A	120.2 (3)	C2B—C1B—C6B	121.0 (3)
C6A—C1A—H1AA	119.9	C2B—C1B—H1BA	119.5
C2A—C1A—H1AA	119.9	C6B—C1B—H1BA	119.5
C3A—C2A—C1A	120.4 (3)	C3B—C2B—C1B	119.8 (3)
C3A—C2A—H2AA	119.8	C3B—C2B—H2BA	120.1
C1A—C2A—H2AA	119.8	C1B—C2B—H2BA	120.1
C4A—C3A—C2A	119.9 (3)	C4B—C3B—C2B	119.3 (3)
C4A—C3A—H3AA	120.0	C4B—C3B—H3BA	120.4
C2A—C3A—H3AA	120.0	C2B—C3B—H3BA	120.4
C3A—C4A—C5A	119.6 (3)	C3B—C4B—C5B	121.2 (3)
C3A—C4A—H4AA	120.2	C3B—C4B—H4BA	119.4
C5A—C4A—H4AA	120.2	C5B—C4B—H4BA	119.4
C4A—C5A—C6A	120.9 (3)	C4B—C5B—C6B	120.1 (3)
C4A—C5A—H5AA	119.6	C4B—C5B—H5BA	119.9
C6A—C5A—H5AA	119.6	C6B—C5B—H5BA	119.9
C1A—C6A—C5A	118.9 (3)	C5B—C6B—C1B	118.6 (3)
C1A—C6A—C7A	119.8 (3)	C5B—C6B—C7B	123.0 (3)
C5A—C6A—C7A	121.3 (3)	C1B—C6B—C7B	118.3 (3)
C8A—C7A—C6A	112.4 (2)	C8B—C7B—C6B	114.6 (2)
C8A—C7A—C14A	111.8 (2)	C8B—C7B—C14B	112.5 (2)
C6A—C7A—C14A	110.6 (2)	C6B—C7B—C14B	109.8 (2)
C8A—C7A—H7AA	107.3	C8B—C7B—H7BA	106.4
C6A—C7A—H7AA	107.3	C6B—C7B—H7BA	106.4
C14A—C7A—H7AA	107.3	C14B—C7B—H7BA	106.4
C9A—C8A—C13A	118.4 (3)	C13B—C8B—C9B	118.5 (3)
C9A—C8A—C7A	124.0 (3)	C13B—C8B—C7B	123.7 (3)
C13A—C8A—C7A	117.6 (3)	C9B—C8B—C7B	117.7 (3)
C8A—C9A—C10A	120.8 (3)	C10B—C9B—C8B	120.7 (3)
C8A—C9A—H9AA	119.6	C10B—C9B—H9BA	119.6
C10A—C9A—H9AA	119.6	C8B—C9B—H9BA	119.6
C11A—C10A—C9A	120.3 (3)	C11B—C10B—C9B	120.3 (3)
C11A—C10A—H10A	119.9	C11B—C10B—H10B	119.8
C9A—C10A—H10A	119.9	C9B—C10B—H10B	119.8
C12A—C11A—C10A	119.4 (3)	C12B—C11B—C10B	119.4 (3)
C12A—C11A—H11A	120.3	C12B—C11B—H11B	120.3
C10A—C11A—H11A	120.3	C10B—C11B—H11B	120.3
C11A—C12A—C13A	120.6 (3)	C11B—C12B—C13B	120.8 (3)
C11A—C12A—H12A	119.7	C11B—C12B—H12B	119.6
C13A—C12A—H12A	119.7	C13B—C12B—H12B	119.6
C12A—C13A—C8A	120.5 (3)	C12B—C13B—C8B	120.2 (3)
C12A—C13A—H13A	119.7	C12B—C13B—H13B	119.9
C8A—C13A—H13A	119.7	C8B—C13B—H13B	119.9
O1A—C14A—N1A	123.1 (3)	O1B—C14B—N1B	123.4 (3)
O1A—C14A—C7A	122.3 (3)	O1B—C14B—C7B	122.4 (2)
N1A—C14A—C7A	114.6 (2)	N1B—C14B—C7B	114.2 (2)
C16A—C15A—C20A	121.0 (3)	C16B—C15B—C20B	120.7 (3)
C16A—C15A—N1A	118.8 (3)	C16B—C15B—N1B	118.9 (3)
C20A—C15A—N1A	120.2 (3)	C20B—C15B—N1B	120.3 (3)
C15A—C16A—C17A	119.8 (3)	C15B—C16B—C17B	120.4 (3)
C15A—C16A—H16A	120.1	C15B—C16B—H16B	119.8
C17A—C16A—H16A	120.1	C17B—C16B—H16B	119.8
C18A—C17A—C16A	120.4 (3)	C18B—C17B—C16B	120.3 (3)
C18A—C17A—H17A	119.8	C18B—C17B—H17B	119.8
C16A—C17A—H17A	119.8	C16B—C17B—H17B	119.8
C17A—C18A—C19A	118.6 (3)	C19B—C18B—C17B	118.4 (3)
C17A—C18A—C21A	121.1 (3)	C19B—C18B—C21B	120.5 (3)
C19A—C18A—C21A	120.3 (3)	C17B—C18B—C21B	121.1 (3)
C20A—C19A—C18A	122.7 (3)	C18B—C19B—C20B	122.8 (3)
C20A—C19A—H19A	118.7	C18B—C19B—H19B	118.6
C18A—C19A—H19A	118.7	C20B—C19B—H19B	118.6
C19A—C20A—C15A	117.5 (3)	C19B—C20B—C15B	117.3 (3)
C19A—C20A—C22A	120.0 (3)	C19B—C20B—C22B	120.5 (3)
C15A—C20A—C22A	122.5 (3)	C15B—C20B—C22B	122.2 (3)
C18A—C21A—H21A	109.5	C18B—C21B—H21D	109.5
C18A—C21A—H21B	109.5	C18B—C21B—H21E	109.5
H21A—C21A—H21B	109.5	H21D—C21B—H21E	109.5
C18A—C21A—H21C	109.5	C18B—C21B—H21F	109.5
H21A—C21A—H21C	109.5	H21D—C21B—H21F	109.5
H21B—C21A—H21C	109.5	H21E—C21B—H21F	109.5
C20A—C22A—H22A	109.5	C20B—C22B—H22D	109.5
C20A—C22A—H22B	109.5	C20B—C22B—H22E	109.5
H22A—C22A—H22B	109.5	H22D—C22B—H22E	109.5
C20A—C22A—H22C	109.5	C20B—C22B—H22F	109.5
H22A—C22A—H22C	109.5	H22D—C22B—H22F	109.5
H22B—C22A—H22C	109.5	H22E—C22B—H22F	109.5
			
C6A—C1A—C2A—C3A	−1.4 (5)	C6B—C1B—C2B—C3B	1.8 (5)
C1A—C2A—C3A—C4A	−0.1 (5)	C1B—C2B—C3B—C4B	−0.5 (5)
C2A—C3A—C4A—C5A	1.3 (5)	C2B—C3B—C4B—C5B	−0.5 (5)
C3A—C4A—C5A—C6A	−1.2 (5)	C3B—C4B—C5B—C6B	0.1 (5)
C2A—C1A—C6A—C5A	1.5 (5)	C4B—C5B—C6B—C1B	1.2 (5)
C2A—C1A—C6A—C7A	−179.1 (3)	C4B—C5B—C6B—C7B	−175.4 (3)
C4A—C5A—C6A—C1A	−0.3 (4)	C2B—C1B—C6B—C5B	−2.2 (5)
C4A—C5A—C6A—C7A	−179.7 (3)	C2B—C1B—C6B—C7B	174.6 (3)
C1A—C6A—C7A—C8A	132.8 (3)	C5B—C6B—C7B—C8B	20.8 (4)
C5A—C6A—C7A—C8A	−47.8 (4)	C1B—C6B—C7B—C8B	−155.8 (3)
C1A—C6A—C7A—C14A	−101.5 (3)	C5B—C6B—C7B—C14B	−107.0 (3)
C5A—C6A—C7A—C14A	77.9 (3)	C1B—C6B—C7B—C14B	76.5 (3)
C6A—C7A—C8A—C9A	111.8 (3)	C6B—C7B—C8B—C13B	−96.5 (3)
C14A—C7A—C8A—C9A	−13.2 (4)	C14B—C7B—C8B—C13B	29.9 (4)
C6A—C7A—C8A—C13A	−69.7 (3)	C6B—C7B—C8B—C9B	84.7 (3)
C14A—C7A—C8A—C13A	165.3 (3)	C14B—C7B—C8B—C9B	−148.9 (3)
C13A—C8A—C9A—C10A	−0.6 (5)	C13B—C8B—C9B—C10B	−0.4 (5)
C7A—C8A—C9A—C10A	177.9 (3)	C7B—C8B—C9B—C10B	178.4 (3)
C8A—C9A—C10A—C11A	−0.1 (6)	C8B—C9B—C10B—C11B	−0.6 (5)
C9A—C10A—C11A—C12A	1.3 (6)	C9B—C10B—C11B—C12B	0.8 (5)
C10A—C11A—C12A—C13A	−1.8 (5)	C10B—C11B—C12B—C13B	0.0 (5)
C11A—C12A—C13A—C8A	1.1 (5)	C11B—C12B—C13B—C8B	−1.1 (5)
C9A—C8A—C13A—C12A	0.1 (5)	C9B—C8B—C13B—C12B	1.2 (4)
C7A—C8A—C13A—C12A	−178.5 (3)	C7B—C8B—C13B—C12B	−177.5 (3)
C15A—N1A—C14A—O1A	−1.3 (5)	C15B—N1B—C14B—O1B	0.5 (5)
C15A—N1A—C14A—C7A	177.7 (3)	C15B—N1B—C14B—C7B	179.5 (3)
C8A—C7A—C14A—O1A	73.9 (3)	C8B—C7B—C14B—O1B	−72.0 (3)
C6A—C7A—C14A—O1A	−52.1 (4)	C6B—C7B—C14B—O1B	56.9 (4)
C8A—C7A—C14A—N1A	−105.2 (3)	C8B—C7B—C14B—N1B	108.9 (3)
C6A—C7A—C14A—N1A	128.8 (3)	C6B—C7B—C14B—N1B	−122.1 (3)
C14A—N1A—C15A—C16A	−109.0 (3)	C14B—N1B—C15B—C16B	109.1 (3)
C14A—N1A—C15A—C20A	71.1 (4)	C14B—N1B—C15B—C20B	−72.1 (4)
C20A—C15A—C16A—C17A	−0.7 (5)	C20B—C15B—C16B—C17B	1.3 (5)
N1A—C15A—C16A—C17A	179.4 (3)	N1B—C15B—C16B—C17B	−179.9 (3)
C15A—C16A—C17A—C18A	0.3 (5)	C15B—C16B—C17B—C18B	−0.8 (5)
C16A—C17A—C18A—C19A	0.6 (5)	C16B—C17B—C18B—C19B	−0.2 (5)
C16A—C17A—C18A—C21A	−178.6 (3)	C16B—C17B—C18B—C21B	178.7 (3)
C17A—C18A—C19A—C20A	−1.2 (5)	C17B—C18B—C19B—C20B	0.7 (5)
C21A—C18A—C19A—C20A	178.0 (3)	C21B—C18B—C19B—C20B	−178.2 (3)
C18A—C19A—C20A—C15A	0.8 (5)	C18B—C19B—C20B—C15B	−0.2 (5)
C18A—C19A—C20A—C22A	−179.1 (3)	C18B—C19B—C20B—C22B	179.9 (3)
C16A—C15A—C20A—C19A	0.2 (4)	C16B—C15B—C20B—C19B	−0.8 (4)
N1A—C15A—C20A—C19A	−179.9 (3)	N1B—C15B—C20B—C19B	−179.6 (3)
C16A—C15A—C20A—C22A	−179.9 (3)	C16B—C15B—C20B—C22B	179.1 (3)
N1A—C15A—C20A—C22A	−0.1 (4)	N1B—C15B—C20B—C22B	0.4 (4)

### Hydrogen-bond geometry (Å, º)

*Cg*1, *Cg*4 and *Cg*5 are the centroids of the C1*A*–C6*A*, C1*B*–C6*B* and C8*B*–C13*B* rings, respectively.

**Table:**

*D*—H···*A*	*D*—H	H···*A*	*D*···*A*	*D*—H···*A*
N1*A*—H1*NA*···O1*B*^i^	0.88 (4)	2.13 (4)	2.980 (3)	163 (3)
N1*B*—H1*NB*···O1*A*	0.86 (5)	2.18 (5)	3.012 (3)	162 (3)
C7*A*—H7*AA*···O1*B*^i^	1.00	2.32	3.258 (4)	155
C9*A*—H9*AA*···O1*A*	0.95	2.48	3.110 (4)	123
C7*B*—H7*BA*···O1*A*	1.00	2.27	3.225 (4)	160
C13*B*—H13*B*···O1*B*	0.95	2.46	3.087 (4)	124
C3*A*—H3*AA*···*Cg*5^ii^	0.95	2.89	3.754 (4)	151
C4*A*—H4*AA*···*Cg*4	0.95	2.84	3.539 (4)	131
C13*A*—H13*A*···*Cg*4^i^	0.95	2.68	3.561 (3)	154
C9*B*—H9*BA*···*Cg*1	0.95	2.62	3.546 (3)	167

Symmetry codes: (i) *x*−1, *y*, *z*; (ii) −*x*+1, −*y*, −*z*+1.
